# Instability Mechanism of Osimertinib in Plasma and a Solving Strategy in the Pharmacokinetics Study

**DOI:** 10.3389/fphar.2022.928983

**Published:** 2022-07-22

**Authors:** Zheng Yuan, Xin Yu, Siyang Wu, Xiaonan Wu, Qiutao Wang, Wenhao Cheng, Weiyu Hu, Chen Kang, Wei Yang, Yingfei Li, Xiao-Yang Zhou

**Affiliations:** ^1^ Center for DMPK Research of Herbal Medicines, Institute of Chinese Materia Medica, China Academy of Chinese Medical Sciences, Beijing, China; ^2^ Department of Oncology, Beijing Hospital, National Center of Gerontology, Institute of Geriatric Medicine, Chinese Academy of Medical Sciences, Beijing, China; ^3^ School of Traditional Chinese Medicine, Shandong University of Traditional Chinese Medicine, Jinan, China; ^4^ School of Chinese Pharmacy, Beijing University of Chinese Medicine, Beijing, China; ^5^ Department of Hepatobiliary Pancreatic Surgery, The Affiliated Hospital of Qingdao University, Qingdao, China; ^6^ The Key Laboratory of Geriatrics, Beijing Institute of Geriatrics, Institute of Geriatric Medicine, Chinese Academy of Medical Sciences, Beijing Hospital/National Center of Gerontology of National Health Commission, Beijing, China

**Keywords:** osimertinib, plasma stability, UPLC-MS/MS, acetonitrile, cysteine, non-small-cell lung cancer

## Abstract

Osimertinib is a third-generation epidermal growth factor receptor tyrosine kinase inhibitor (EGFR-TKI) and a star medication used to treat non-small-cell lung carcinomas (NSCLCs). It has caused broad public concern that osimertinib has relatively low stability in plasma. We explored why osimertinib and its primary metabolites AZ-5104 and AZ-7550 are unstable in rat plasma. Our results suggested that it is the main reason inducing their unstable phenomenon that the Michael addition reaction was putatively produced between the Michael acceptor of osimertinib and the cysteine in the plasma matrix. Consequently, we identified a method to stabilize osimertinib and its metabolite contents in plasma. The assay was observed to enhance the stability of osimertinib, AZ-5104, and AZ-7550 significantly. The validated method was subsequently applied to perform the pharmacokinetic study for osimertinib in rats with the newly established, elegant, and optimized ultra-performance liquid chromatography–tandem mass spectrometer (UPLC-MS/MS) strategy. The assay was assessed for accuracy, precision, matrix effects, recovery, and stability. This study can help understand the pharmacological effects of osimertinib and promote a solution for the similar problem of other Michael acceptor-contained third-generation EGFR-TKI.

## 1 Introduction

Non-small-cell lung cancer (NSCLC) is the largest pulmonary carcinoma subgroup of lung cancer and belongs to the most frequent malignant diseases (Xue et al., 2011; Gridelli et al., 2015). Also, the popular tyrosine kinase inhibitor (TKI) application has been well testified to benefit NSCLC patients with epidermal growth factor receptor (EGFR) mutations (Ke and Wu, 2016; Zhou et al., 2020). Osimertinib is a third-generation EGFR-TKI, selectively inhibits mutated EGFR (e.g., EGFR with exon 19 deletion, L858R, T790M, G719X, L861Q, and S768I), and has recently become the prominent star medicine for treating EGFR-mutated patients reaching a median progression-free survival (PFS) of 18.9 months (Remon et al., 2018; Soria et al., 2018; Cho et al., 2020; Floc’h et al., 2020). In addition, apart from the role of osimertinib as an anticancer drug, it has been found as a promising adjuvant agent to sensitize drug-resistant cancer cells to chemotherapy (Barbuti et al., 2019). Therefore, the analysis of osimertinib and its metabolites may promise better profiling of the distribution of components and enhance the understanding of the pharmacodynamics effects and toxicity of drugs in pre-clinic and clinic trials, and as a result in promoting osimertinib-related new drug research and development.

The osimertinib analysis in the matrix such as human serum, human plasma, human urine, rat plasma, and mouse brain has been constructed through liquid chromatography–tandem mass spectrometric (LC-MS) assay (Ballard et al., 2016; Rood et al., 2016; Irie et al., 2019; Mitchell et al., 2019; Fresnais et al., 2020; Xiong et al., 2022). However, the instability of osimertinib in plasma and whole blood at an ambient temperature drew attention recently. Rood et al. (2016) measured osimertinib in human plasma (either lithium heparin- or sodium EDTA-treated) stored at −30°C by salting-out assisted liquid–liquid extraction (LLE) using acetonitrile and magnesium sulfate (Rood et al., 2016). However, Veerman et al. (2019) found that the mass response of osimertinib decreased after incubation of plasma with osimertinib, whether at an ambient or higher temperature, thus pointing that both blood and plasma samples should be kept and processed solely on ice. This harsh environment may be uncomfortable for laboratory operators. In addition, Veelen et al. (2020) validated the sample preparation of human plasma containing osimertinib. Even if they pointed out that acidification of serum samples enhanced the stability of osimertinib, a lower temperature under 2°C was needed whether for storing or preparing the samples. A method is severely needed for storing and operating plasma containing osimertinib. Hence, a practical, convenient, and universal approach is urgently required for plasma preparation to keep the stability of osimertinib.

**FIGURE 1:**
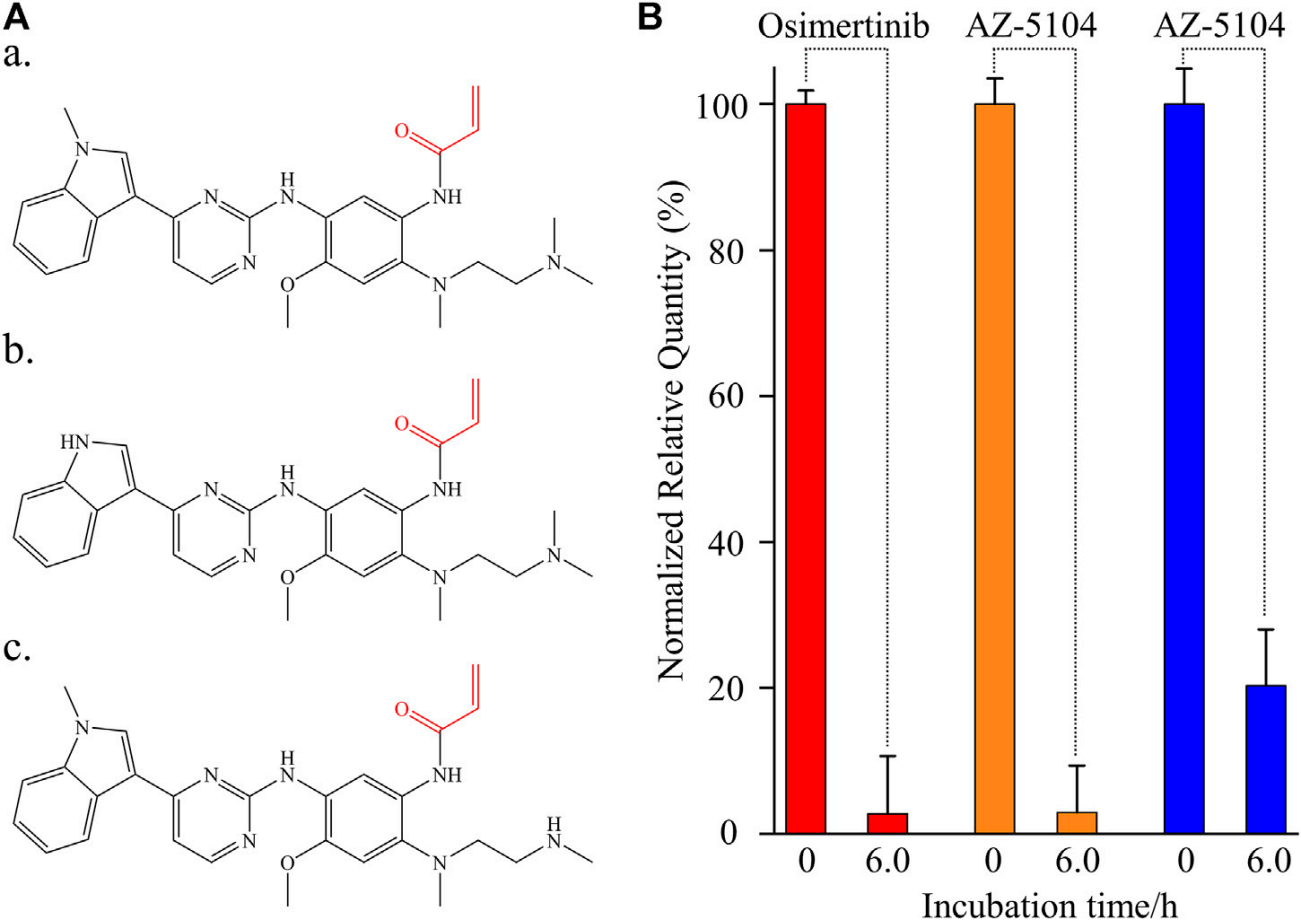
Instability of osimertinib, AZ-5104, and AZ-7550 in rat plasma. **(A)** Chemical structure of **(a)** osimertinib, **(b)** AZ-5104, and **(c)** AZ-7550. The Michael acceptor functional groups are labelled red. **(B)** Histogram of the normalized relative quantity of osimertinib (red), AZ-5104 (orange), and AZ-7550 (blue) after the incubation in rat plasma at 39°C for 0 and 6 h. The concentrations of analytes including propranolol in rat plasma were 10 ng/ml. The normalized relative quantity (NRQ) was calculated as the analyte/propranolol area ratio corresponding to the proposed incubation time of 0 h or 6 h over the area ratio for 0 h (*n* = 6). If not specified, the conditions listed upon adapted to the follow-up experiments.

In the perspective of organic chemistry, whether osimertinib or its circulating metabolites, AZ-5104 and AZ-7550, contain the same Michael acceptor responsible for the covalent reaction with the cysteine-797 residue in the ATP-binding site of the EGFR kinase *in vivo* (Figure 1A) (Wu and Shih, 2018; Lategahn et al., 2019; Gao et al., 2022). Coincidentally, Dickinson et al. (2016) found that the remaining osimertinib decreased in the plasma of mice, rats, dogs or humans at 37°C for 6 h. Significantly, they found that human serum albumin (HSA) contributes to decreasing osimertinib during incubation. To the best of our understanding, serum albumin is the major protein component in mammal plasma (Minchiotti et al., 2008). In addition, we also noticed that the total cysteine concentration in human plasma is as high as 38,650 ng/ml (Wang et al., 2018). The evidence reviewed here suggests that a pertinent role of free or non-free cysteine in plasma may induce the reduction of osimertinib during the related investigation.

This study seeks to understand and explain that cysteine’s potential role in plasma may affect the stability of osimertinib, AZ-5104, and AZ-7550 during the storage of plasma samples at room or higher temperatures. A strategy was applied to keep osimertinib stability by directly adding acetonitrile during preparation in plasma. In addition, we studied the oral pharmacokinetic study in five Sprague–Dawley (SD) rats using our newly validated UPLC-MS/MS coupling stability strategy. In general, we successfully set up an available and reliable method to increase osimertinib stability and apply it to the osimertinib pharmacokinetics study in rats.

## 2 Materials and Methods

### 2.1 Chemicals, Drugs, and Equipment

Osimertinib (purity 99.9%), AZ-5104 (purity 99.7%), and AZ-7550 hydrochloride (purity 99%) standards were purchased from MedChemExpress (Monmouth Junction, NJ, United States). Propranolol was used as inner standard (IS) during UPLC-MS/MS analysis and was bought from Sigma-Aldrich (Dorset, United Kingdom). Acetonitrile (ACN), formic acid (FA), methanol (MeOH), isopropanol (IPA), and dimethyl sulfoxide (DMSO) were all analytical reagents (ARs) provided by Merck (Darmstadt, Germany). L-Cysteine (Cys, 99%) was purchased from Bailingke (Haidian, Beijing, China). A TAGRISSO^®^ (osimertinib) tablet (80 mg) produced by AstraZeneca (United States) was obtained from Beijing Hospital. Sodium carboxymethyl cellulose (CMC·Na) was supplied by Tianjin Chemical Reagent Company (Tianjin, China). 1 × PBS (0.01 M, pH 7.2–7.4, cell culture) was obtained from Solarbio (Beijing, China). Ethylenediaminetetraacetic acid disodium salt (EDTA·2Na) was purchased from National Pharmaceutical Group Chemical Reagent Company (Beijing, China). Male Sprague–Dawley (SD) rats (weighed 180–200 g) were provided by Beijing Vital River Laboratory Animal Technology Co., Ltd. (Beijing, China). All solutions were prepared using a Milli-Q system (Millipore, Massachusetts, United States) with an electrical resistivity of 18.2 MΩ except for L-cysteine using 1 × PBS instead. All solutions were filtered through a 0.45-mm filter. Two kinds of centrifugal filter units with a molecular weight cut-off (MWCO) of 3.0 kDa and 30 kDa were also bought from Millipore. An electronic thermostatic mixing water bath pot (DFD-700) was provided by Zhongxing (Beijing, China).

### 2.2 UPLC-MS/MS Instrumentation Setting and Operating Conditions

An AB Sciex API 5500 QTrap mass spectrometer (Toronto, Canada) interfaced with a Waters Acquity UPLC separation module was used to detect and quantify osimertinib, AZ-5104, AZ-7550, and propranolol. Empower 3.0 was used in UPLC and Analyst 1.6.2 software in the mass spectrometer. Other settings for mass spectrometry parameters of the four compounds are shown in Table 1.

**TABLE 1:** Optimized MRM parameters of the analytes and IS in this study.

Analyte	Retention time (min)	Precursor ion species	MRM transition	Dp (V)	Ce (eV)
			Precursor ion→product ion (m/z)		
Propranolol	2.23	[M + H]^+^	260.1→116.0	140	23
Osimertinib	2.02	[M + H]^+^	500.2→72.1	80	60
AZ-5104	1.84	[M + H]^+^	486.4→72.1	80	55
AZ-7550	1.97	[M + H]^+^	486.3→429.3	80	32

Chromatographic separation was achieved on a Waters Acquity UPLC HSS T3 1.8 μm column (2.1 mm × 50 mm) at 40°C using a mobile phase containing 0.1% FA that consisted of solvent A (water) and solvent B (ACN). The mobile phase was delivered at 0.3 ml/min, and an elegant gradient program was used as follows: 0–0.5 min, at 10% solvent B; 0.5–2.0 min, from 10 to 90% solvent B; 2.0–2.5 min, at 100% solvent B; and 2.5–3.0 min, at 10% solvent B. To eliminate carryover as much as possible, the injection needle was washed with 2.5 ml strong needle wash solution [ACN–MeOH–IPA–water (25:25:25:25, v/v) and 2.5 ml weak needle wash solution (FA–ACN–water (0.5:50:50, v/v)] before each injection.

### 2.3 Sample Preparation

For researching drug–plasma interaction and validating the UPLC-MS/MS method, enough blank SD rat plasma must be prepared before relative experiments. First, about 200 ml of rat blood was extracted with 5.0 mM EDTA·2Na from the abdominal aorta. Second, the blood was centrifuged at 4.0°C at 12,000 rpm for 5.0 min. The plasma (upper layer) was then aliquoted in a 1.0-ml/tube and stored at −70°C until use.

About 10 ml of rat plasma was added into one centrifugal filter unit as molecular weight cut-off in need and filtered by centrifuging for 40 min at 5,000 rpm at 4°C. The filtered-out liquid turned transparent and was stored at −70°C until use.

Osimertinib, AZ-5104, AZ-7550, and propranolol powders were dissolved to 1.0 mg/ml by an appropriate amount of DMSO as a stock solution, aliquoted in a 100-μl/tube and stored at −70°C until use. Working solutions were prepared extemporaneously by diluting stock solutions with 50% acetonitrile.

The L-cysteine powder was fast dissolved to 1.0 mg/ml by 1 × PBS as storage solution and quickly stored at −70°C as soon as possible. To inhibit the spontaneous dimerization of cysteine to cystine in PBS, the cysteine solution was freshly prepared before each experiment.

An osimertinib tablet was ground into a powder using a mortar and pestle. Then, a certain mass of powder samples converted into 8.0 mg osimertinib was weighed and added into 10 ml 12.5 mM CMC·Na. The sample was subjected to vortex mixing (3.0 min), sonicated (3.0 min), and vortex mixing again. The vortex–sonication cycle was repeated three times.

### 2.4 Plasma/Cysteine–Drug Incubation

Generally, each 45 μl L-cysteine solution with a certain concentration (diluted by 1 × PBS) or rat plasma was added with 5.0 μl working solution and mixed using vortex apparatus. Then, 5.0 μl propranolol with a 100 ng/ml concentration was added and mixed as described previously. The mixture was incubated at 37°C in a thermostatic mixing water bath pot for a certain period of time. Afterward, the tube was added with 350 μl ACN, vortexed (room temperature, 2000 rpm, 5.0 min), sonicated (4°C, 5.0 min), and centrifuged (4°C, 12,000 rpm, 5.0 min). Then, 200 μl upper liquid was taken into sample tubes for UPLC-MS/MS analysis.

### 2.5 UPLC-MS/MS Validation

The linearity, accuracy, precision, selectivity, specificity, matrix effect, recovery, and stability were all experimentally validated. The detailed validation process descriptions are as follows:

#### 2.5.1 Linearity

Seven calibration standards samples with sequential concentrations of 1.0 (lower limit of quantitation, LLOQ), 3.0, 9.0, 27, 81, 243, and 729 ng/ml for osimertinib, AZ-5104, and AZ-7550 in rat plasma with additional 10 ng/ml propranolol (inner standard, IS) were made up in three successive batches. Calibration curves were obtained by plotting each analyte’s peak area ratio to IS versus the nominal concentration of calibration standards. The weighting factor was set at 1/x^2^. The deviations from the mean for each calibration standard should be within ± 15%. The LLOQ should not exceed ± 20%.

#### 2.5.2 Specificity and Selection

The specificity of the method was assessed by analyzing six batches of blank SD rat plasma to validate the interference induced by chromatographic conditions at each analyte’s retention time. The criteria of acceptance for the method specificity and selectivity are that the peak areas in the double blank samples should be less than 20% of the peak areas of LLOQ samples in each batch.

#### 2.5.3 Accuracy and Precision

Three standards samples with sequential concentrations of 2.0, 27, and 583 ng/ml for osimertinib, AZ-5104, and AZ-7550 in rat plasma with additional 10 ng/ml propranolol (inner standard, IS) were set as the low-quality control (LQC), middle-quality control (MQC), and high-quality control (HQC), respectively. The intra- and inter-batch precision was indicated as the percent relative standard deviation (RSD%). The intra- and inter-batch accuracy was indicated as the nominal concentration’s relative error (RE). Both precision and accuracy were determined by analyzing six replicates of QC in three consecutive batches. The acceptance criteria of precision for the intra- and inter-batch of QC should be within ± 15%, except ± 20% for LLOQ. The acceptance criteria for the intra- and inter-batch accuracy are that the RE of QC should be within 85–115% of their nominal concentration at each QC concentration except 80–120% for LLOQ.

#### 2.5.4 Matrix Effect and Recovery

The degree of the matrix influencing each analyte’s tandem mass spectrum signal was assessed by comparing the peak area of the analyte spiked after extraction and the peak area of standard in neat solution at each QC concentration using six batches of the blank matrix from different sources. The matrix effect’s acceptance criteria are that the coefficient of variation of the IS-normalized matrix effect calculated from the six batches of the matrix should be within 85–115%. The relative recovery was determined by comparing each extracted sample’s obtained value with the extracts of the spiked blank matrix with the analyte post-extraction at three QC concentration levels.

#### 2.5.5 Stability

Each QC analyte’s long-term and freeze-thaw stability in the SD rat plasma matrix with the addition of ACN (350 μl) for osimertinib was evaluated. To assess the analyte’s long-term stability, all QC samples were preserved at −70 C for 2 months before sample processing and analyzing whether ACN was pre-added or not. For assessing the analyte’s freeze–thaw stability, all QC samples were processed three cycles of freezing (−70 C) and thawing at room temperature for 1.0 h before sample processing and analyzing, ignoring the addition of ACN. The acceptance criteria were less than 15% of the nominal values for accuracy and within 15% for precision.

### 2.6 Pharmacokinetics Study

Five male SD rats (180–200 g) were fed autoclaved standard laboratory food and free to access sterile water and kept in an environmentally controlled breeding room (temperature, 20 ± 2°C; humidity, 60 ± 5%; 12-h dark/light cycle) for at least 6 days before experimentation. All rats were fasted for 12 h before the experiment but were allowed free access to water. The rats were given osimertinib dissolved by CMC·Na orally at 8.0 mg/kg. The rats were anesthetized by isoflurane at 0.08, 0.25, 0.5, 1.0, 2.0, 4.0, 6.0, 8.0, 10, and 24 h after oral administration. Blood samples (about 200 μl) were immediately collected from an eye socket vein and preserved in EDTA tubes. Then, the blood sample was centrifuged in an Eppendorf laptop centrifuge 5417C (Hamburg, Germany) for 5.0 min at 12,000 rpm to obtain plasma. Plasma with a volume of 50 μl was transferred into a tube added with 350 μl ACN beforehand. The mixture was quickly vortexed by an MS3 basic vortex mixer (IKA GmbH, Germany) at 2000 rpm for 2.0 min and stored at −70°C until further processed.

Animal welfare and experimental procedures strictly followed the Guide for the Care and Use of Laboratory Animals (The Ministry of Science and Technology of China, 2006) and the related ethical regulations of the China Academy of Chinese Medical Sciences (CACMS).

## 3 Results and Discussion

### 3.1 The Instability of Osimertinib/AZ-5104/AZ-7550 in Rat Plasma

The stability of osimertinib, AZ-5104, and AZ-7550 was investigated by observing the relative quantitative change after incubating each 10 ng/ml chemical with 50 μl rat plasma (*n* = 6) for 0 and 6.0 h. For a better description, the three negative control experiments (incubation time = 0) were used for normalization (Figure 1B). After 6.0 h of incubation, the detected relative quantity of osimertinib, AZ-5104, and AZ-7550 fell sharply to 2.76%, 2.96%, and 20.3%, respectively, strongly showing severe instability of the three chemicals in rat plasma. These phenomena are consistent with a previous study using human plasma as a warm bath matrix (Dickinson et al., 2016), thus excluding the possibility induced by species differences. Then, the instability of all three chemicals should be attributed to the typical chemical structure of all three drugs and the material basis of animal plasma.

Similar to other third-generation anti-tumor-targeted drugs, osimertinib contains two primary functional groups. One is the skeleton which can recognize the ATP-binding site of the EGFR kinase (To et al., 2019), and the other is the Michael acceptor (red part of Figures 1A, a), which can react with the cysteine-797 residue of the EGFR kinase to form irreversible covalent bond formation. AZ-5104 and AZ-7550 are the primary demethylated metabolites of osimertinib (Jiang et al., 2018), sharing the same active Michael acceptor with osimertinib (red part of Figures 1A–C). The instability of all three chemicals is likely due to the Michael addition reaction deriving from the Michael acceptor and the cysteine in the plasma matrix. Considering that the high concentration of total cysteine in plasma is elevated to 10 μM and the pharmacology of osimertinib, we propose that the protein-containing cysteine residue and free cysteine in plasma are significant factors causing the decline of osimertinib, AZ-5104, and AZ-7550 in plasma.

### 3.2 Protein and Free Cysteine Reduce the Stability of Osimertinib/AZ-5104/AZ-7550

**FIGURE 2:**
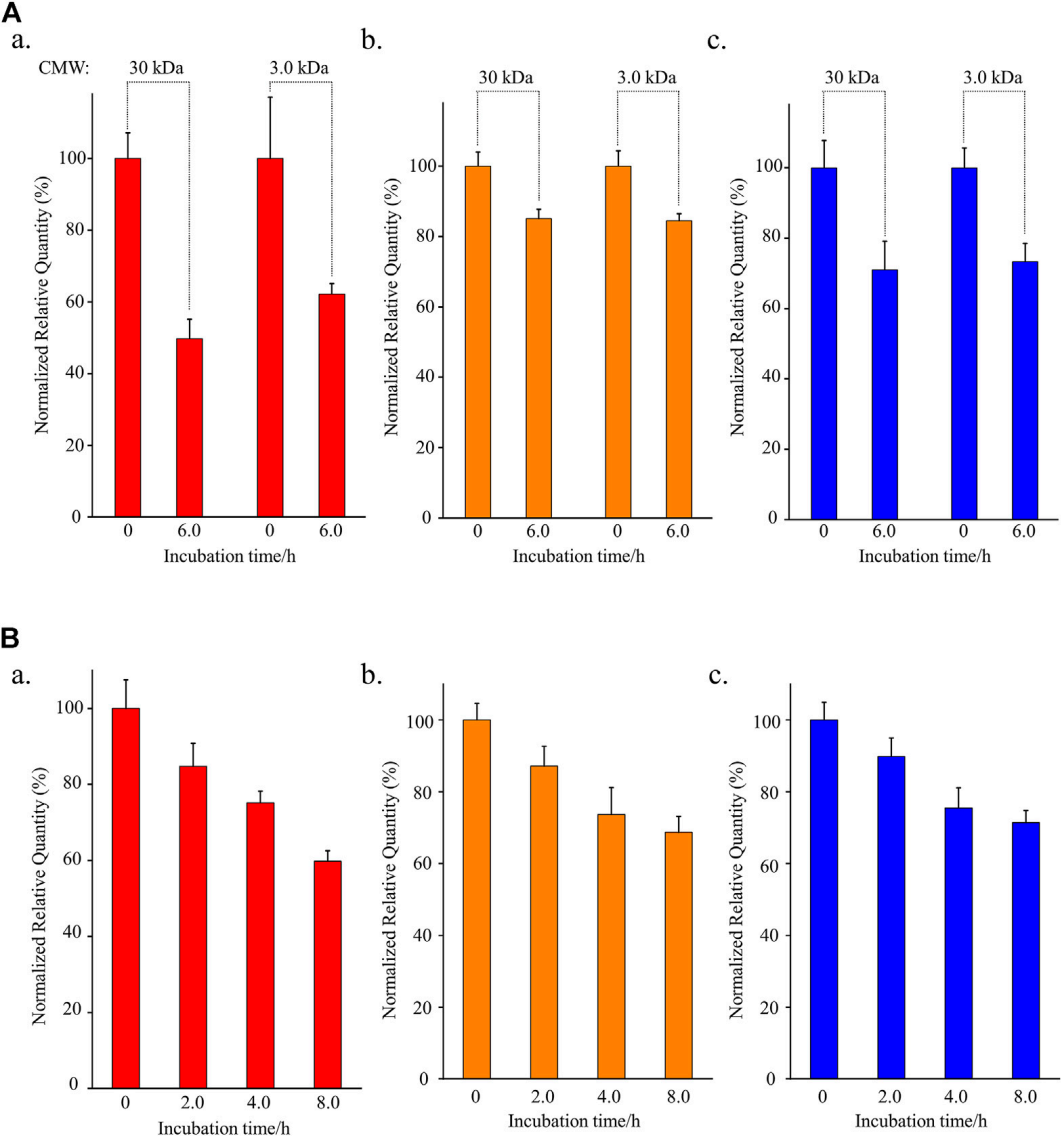
Investigation of the stabilities of (a) osimertinib (red), (b) AZ-5104 (orange), and (c) AZ-7550 (blue) in **(A)** filtered rat plasma and **(B)** L-cysteine. In **(A)**, the cut-off molecular weight (CMW) was 30 kDa or 3.0 kDa. In **(B)**, L-cysteine concentration was 10.0 μg/ml. Note: the working solutions except for L-cysteine were kept at 39°C for the longest incubation time to eliminate all four chemicals’ temperature instability.

To test the contribution of plasma protein in the instability of osimertinib, AZ-5104, and AZ-7550, rat plasma filtered by a centrifugal filter unit with a specific molecular weight cut-off (MWCO) was used to incubate with the three chemicals, respectively, instead of untreated plasma. The centrifugal filter unit was used to cut off macromolecule proteins above 30 kDa or 3.0 kDa from plasma. The normalized relative quantity of osimertinib was 49.8% and 62.2% (Figure 2A, a) after incubating with plasma treated by the centrifugal filter with MWCO 30 kDa or 3.0 kDa for 6.0 h, respectively. Furthermore, the values were 85.1% and 84.5% for AZ-5104 (Figures 2A,B) and 71.0% and 73.3% for AZ-7550 (Figures 2A,C). Through comparing with the detected osimertinib, AZ-5104, and AZ7550 using untreated plasma as substrates (Figure 1B), protein-filtered plasma enhanced the stability of osimertinib, AZ5104, and AZ7550. Therefore, macromolecular protein in plasma is one of the main factors inducing the instability of osimertinib, AZ-5104, and AZ-7550 in plasma.

To further identify the influence of free cysteine on the stability of osimertinib, AZ-5104, and AZ-7550, another experiment that incubates 10 μg/ml cysteine aqueous solution with 10 ng/ml chemical substrate for 2.0, 4, 0, and 8.0 h was designed. In addition, PBS with a pH of 7.2–7.4 was used as a solvent to mimic the rat plasma’s pH environment. The detected osimertinib, AZ-5104, and AZ-7550 present a highly negative relationship with the incubation duration (Figures 2A,B,C), and the normalized relative quantity was 59.8%, 68.7% and 71.4%, respectively, after 8.0 hours of incubation.

It is well known that only 20 amino acids are involved in protein synthesis in mammals, in which there is only one amino acid containing biological thiol, cysteine. However, osimertinib, carrying α, β-unsaturated carbonyls, was designed to be able to react with biological thiols, resulting in irreversible small-molecule-thiol adducts. No amino acid can react with osimertinib, AZ-5104, and AZ-7550 except for cysteine. However, homocysteine, an intermediate product of methionine metabolism in mammals (Kumar et al., 2017), contains one sulfhydryl group, can react with the Michael acceptor of osimertinib, AZ-5104, and AZ-7550. The average homocysteine concentration in mammals such as human is 10 μM (Brouwer et al., 1998), much lower than that of cysteine (319 μM) (Wang et al., 2018). Overall, these results indicate that the protein and free cysteine in plasma are the main factors inducing the instability of osimertinib in plasma.

### 3.3 Acetonitrile Maintains the Stability of Osimertinib in Plasma

In general, adding an appropriate additive is an efficient strategy to promise the stability of chemicals in a matrix. For example, adding enough complexing agent such as EDTA can enhance the stability of drugs and foods in plasma (Keowmaneechai and McClements, 2002; Song et al., 2014). This strategy is based on the fact that a complexing agent can decrease the quantity of free metal ions, which is the cofactor to the protein that can degrade the chemical. In addition, the incubation of proteinase K can also enhance the stability of several chemicals in plasma. Such a method is based on proteinase K that can disrupt the three-dimensional structure of proteins in plasma which degrades the target drug (Kim et al., 2019). However, based on our findings, the instability of osimertinib in plasma is due to protein and the free small organic molecule cysteine. As a result, both strategies targeting reducing protein activity were limited in overcoming the effect of free cysteine or cysteine residue in the plasma matrix. In addition, phenylmethylsulfonyl fluoride (PMSF), sodium fluoride (NaF), and diisopropyl fluorophosphate (DFP) were also applied to improve the stability of osimertinib, AZ-5104, and AZ-7550 in rat plasma based on the previous experiment. However, we did not achieve the desired results (data not shown). Therefore, a new additive is urgently needed to breach such barrier based on the chemical or physical theory.

**FIGURE 3:**
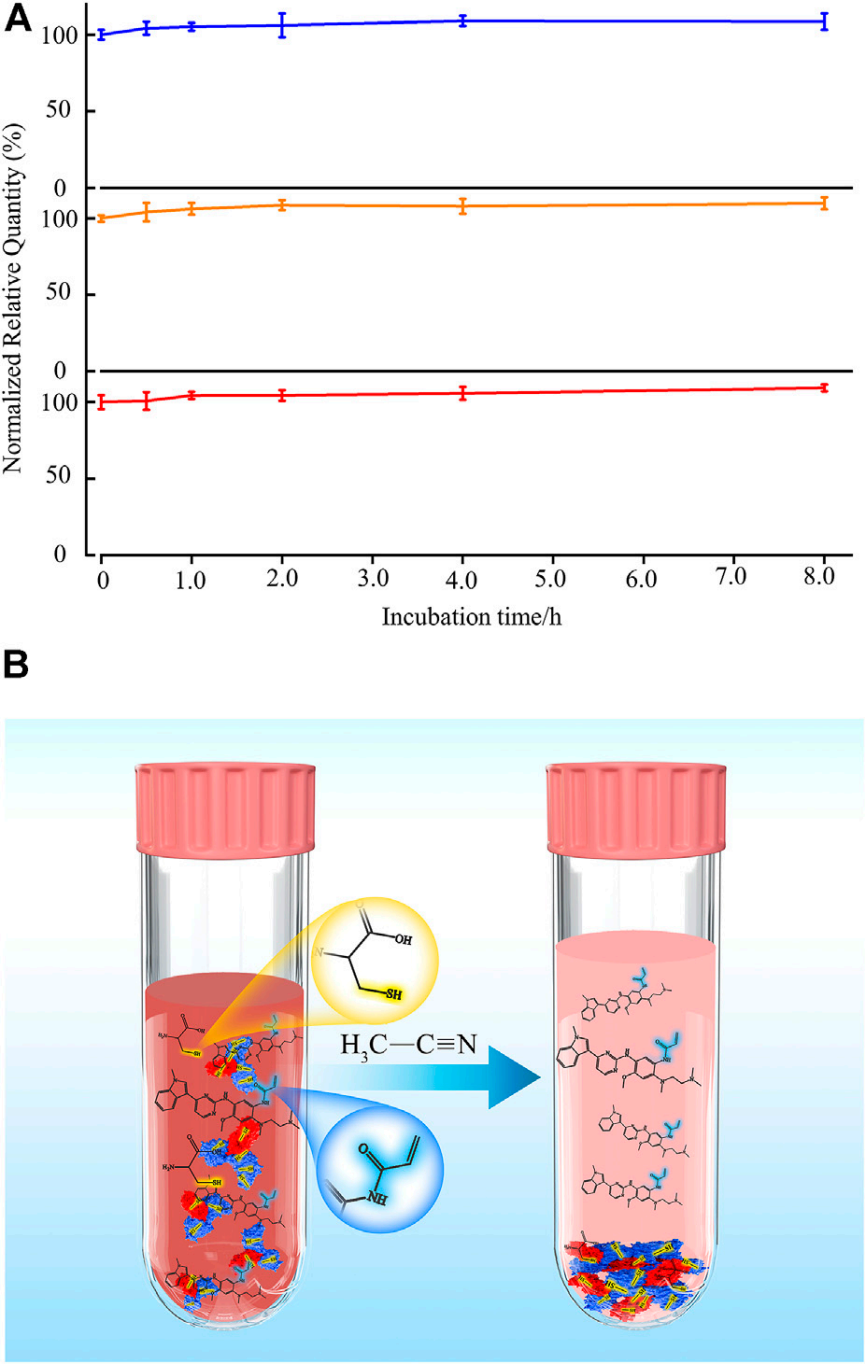
**(A)** Addition of acetonitrile keeps the stability of osimertinib (blue), AZ-5104 (orange), and AZ-7550 (red) in rat plasma. Each 5.0 μl chemical substrate (100 ng/ml) was incubated with 45 μl rat plasma and vortexed for 1.0 min in the experiment. Then, 350 μl acetonitrile was added, and 400 μl solution was vortexed at 1,600 rpm for 5.0 min and incubated at 39°C for 0.5, 1.0, 2.0, 4.0, and 8.0 h before the mass spectrometry sample preparation and measurement. **(B)** Schematic representation of adding acetonitrile in plasma protects third-generation EGFR-TKI osimertinib and its main metabolites from Michael addition with free cysteine and protein.

Organic solvents such as acetonitrile, methanol, isopropanol, and acetone are primary solvents used to precipitate protein in the plasma sample (Bruce et al., 2009). Interestingly, the solubility of cysteine in acetonitrile is the lowest among other solvents (Han et al., 2020), indicating that acetonitrile is an appropriate additive for precipitating both protein and cysteine in plasma. To test this hypothesis, the stability of osimertinib in plasma while adding acetonitrile at 39 C (rat temperature *in vivo*) was tested (Figure 3A). Excitingly, no substantial changes were noticed in osimertinib levels even under incubation for 8.0 h (Figure 3, line in blue). Moreover, the same phenomenon was also observed using AZ-5104 and AZ-7550 as chemical substrates (Figure 3, lines in orange and red, respectively). Apparently, acetonitrile is an effective stabilizer for osimertinib in plasma (Figure 3B).

### 3.4 Method Validation

**FIGURE 4:**
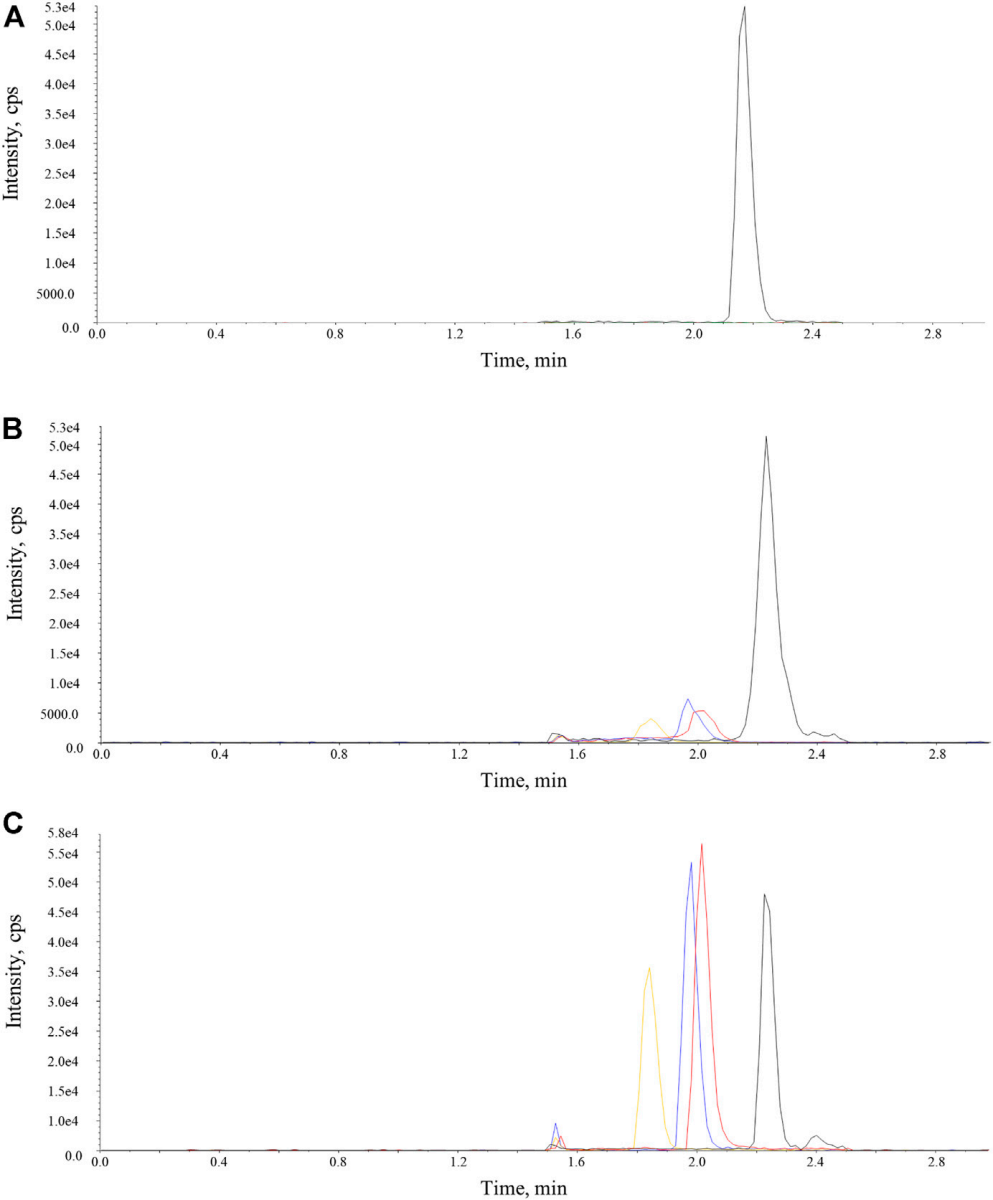
Representative chromatograms of osimertinib, AZ-5104, AZ-7550, and IS in SD rat plasma samples. The red, orange, and blue lines represent osimertinib, AZ-5104, and AZ-7550. The black line represents IS. **(A)** Blank, **(B)** LLOQ, and **(C)** real SD rat plasma sample.

On the basis of aforementioned results, a systemic method validation for measuring osimertinib, AZ-5104, and AZ-7550 assisted by adding acetonitrile was performed according to guidelines set by Pharmacopoeia of the People’s Republic of China (2020) and the US Food and Drug Administration (2013). The UPLC-MS/MS condition was first optimized and four samples including blank, LLOQ, and plasma, were separated and detected successfully (Figure 4), showing no significant interference from SD rat plasma observed at the retention time of analytes and IS. In addition, the representative calibration curve equation was y = 0.00941x + 0.00158 (*r* = 0.9987) to osimertinib, y = 0.00758x + 0.000415 (r = 0.9983) to AZ-5104, and y = 0.0102x + 0.00235 (*r* = 0.9978) to AZ-7550. The accuracy and intra- and inter-day precision of osimertinib, AZ-5104, and AZ-7550 in SD rat plasma were calculated and summarized as given in Tables 2A–C, respectively. The data of extraction recovery and matrix effect obtained are presented in Table 3. In particular, the long-term stability and freeze–thaw stability in the SD rat plasma matrix with the addition of ACN were studied, and the results are summarized in Table 4, indicating that acetonitrile keeps the stability of osimertinib in plasma efficiently.

**TABLE 2:** Accuracy and intra- and inter-day precision of **(A)** osimertinib, **(B)** AZ-5104, and **(C)** AZ-7550 in SD rat plasma (*n* = 6). **(A)**

Nominal concentration (ng/ml)		Measured concentration (ng/ml) (mean ± SD)	Accuracy (%)	Intra-day precision (CV)	Inter-day precision (%) (CV)
2.0	Day 1	1.83 ± 0.09	91.5	4.9	6.0
	Day 2	2.07 ± 0.05	103.5	2.4	
	Day 3	1.92 ± 0.08	96.0	4.2	
27	Day 1	26.30 ± 1.00	97.4	3.8	4.2
	Day 2	27.65 ± 0.81	102.4	2.9	
	Day 3	28.53 ± 0.58	105.7	2.0	
583	Day 1	570.53 ± 13.40	97.9	2.3	2.2
	Day 2	572.33 ± 13.60	98.2	2.4	
	Day 3	565.17 ± 10.03	96.9	1.8	

Nominal concentration (ng/ml)		Measured concentration (ng/ml) (mean ± SD)	Accuracy (%)	Intra-day precision (CV)	Inter-day precision % (CV)
2.0	Day 1	2.11 ± 0.15	105.5	7.1	6.6
	Day 2	1.96 ± 0.06	98.0	3.1	
	Day 3	1.89 ± 0.03	94.5	1.6	
27	Day 1	26.3 ± 0.83	97.4	3.2	3.6
	Day 2	27.50 ± 0.89	101.9	3.2	
	Day 3	26.63 ± 0.93	98.6	3.5	
583	Day 1	564.50 ± 13.31	96.8	2.4	3.1
	Day 2	575.00 ± 21.81	98.6	3.8	
	Day 3	566.50 ± 22.43	97.2	4.0	

Nominal concentration (ng/ml)		Measured concentration (ng/ml) (Mean ± SD)	Accuracy (%)	Intra-day precision (CV)	Inter-day precision % (CV)
2.0	Day 1	1.92 ± 0.15	96.0	7.8	7.8
	Day 2	2.13 ± 0.16	106.5	7.5	
	Day 3	1.93 ± 0.07	96.5	3.6	
27	Day 1	26.32 ± 0.75	97.5	2.8	3.6
	Day 2	26.11 ± 0.93	96.7	3.6	
	Day 3	27.55 ± 0.51	102.0	1.9	
583	Day 1	566.17 ± 14.57	97.1	2.6	3.3
	Day 2	560.17 ± 11.48	96.1	2.0	
	Day 3	597.50 ± 12.00	102.5	2.0	

**TABLE 3:** Matrix effect and recovery of osimertinib, AZ-5104, and AZ-7550 in SD rat plasma (*n* = 6).

	Nominal concentration (ng/ ml)	Matrix effect (%)	Recovery (%)
Osimertinib	2.0	93.5	90.2
	27	98.4	93.2
	583	96.3	92.5
AZ-5104	2.0	90.1	96.7
	27	103.2	93.3
	583	96.3	95.0
AZ-7550	2.0	95.6	91.2
	27	105.3	94.7
	583	103.9	90.2

**TABLE 4:** Stability of osimertinib in SD rat samples (*n* = 6).

	Nominal concentration (ng/ml)	Measured concentration (ng/ml) (mean ± SD)	Accuracy (%)	RSD (%)
Long-term stability	2.0	1.92 ± 0.10	96.00	5.2
	27	28.12 ± 0.83	104.15	3.0
	583	565.50 ± 17.82	97.00	3.2
Freeze–thaw stability	2.0	1.93 ± 0.14	96.50	7.3
	27	25.72 ± 1.69	95.26	6.6
	583	570.33 ± 18.96	97.83	3.3

### 3.5 Pharmacokinetics Study of Osimertinib in Rat Plasma

**FIGURE 5:**
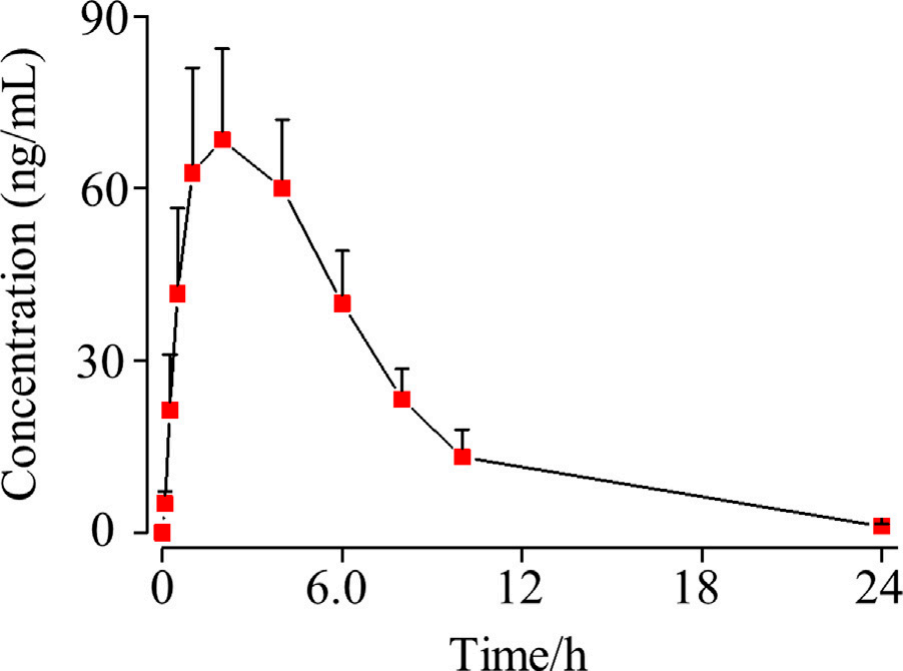
SD rat plasma concentration–time curve of osimertinib (mean ± standard deviation, *n* = 5) after oral administration.

To further apply such an assay in a pre-clinical pharmacokinetics study, we performed pharmacokinetics study of osimertinib in rat plasma using five male SD rats. Unlike the conventional pre-clinical pharmacokinetics experimental scheme, the preparation of rat plasma was online. The rat blood was taken from the eye socket vein at the appropriate time point and immediately followed by high-speed centrifugation for plasma preparation. Then, 50 μl of freshly made rat plasma was mixed with a seven-order volume of acetonitrile for maintaining the stability of osimertinib. Each concentration of osimertinib in rat plasma was obtained by the validated UPLC-MS/MS assay, and all data are described statistically as a concentration–time curve (Figure 5), displaying the concentration of osimertinib in rat plasma peaks (65.5 ng/ml) after 2.5 h of oral administration which slowly reduced in the following 20 h. Also after 24 h of oral administration, osimertinib was not detectable in rat plasma. Such a pre-clinical experiment indicates the applicability of the strategy that stabilizes osimertinib in plasma by adding ACN and the methodology in processing rat plasma online.

One should note that acetonitrile treatment should be concomitant with the whole animal experiment before sample storage, thus adding tension and complexity to a certain extent. Therefore, an optimal and feasible protocol is needed to make the proposed strategy convenient for operation in future research applications.

## 4 Conclusion

In summary, we confirmed that both the protein and cysteine in plasma are the main factors that induce the instability of osimertinib and its primary metabolites AZ-5104 and AZ-7550 through a series of experiments. A new strategy stabilizing osimertinib by adding enough acetonitrile was proposed and validated based on the physicochemical properties of plasma and cysteine. In addition, coupled with the online biological sample processing method, the strategy was successfully applied in a pre-clinical pharmacokinetics study. The proposed strategy adapts to human subjects, and a relative work is underway. It is notable that most third-generation EGFR-TKIs (such as naquotinib and olmutinib) contain at least one Michael acceptor targeting cysteine whether free in plasma or imported into the protein (Attwa et al., 2018; Alrabiah et al., 2019). Therefore, the organic additive-assisted method coupled with the online biological sample processing methodology may promote the research and development of other third-generation EGFR-TKI in the future study.

## Data Availability

The original contributions presented in the study are included in the article/Supplementary Material. Further inquiries can be directed to the corresponding authors.

## Abbreviations

ACN, Acetonitrile; AR, analytical reagent; DMSO, dimethyl sulfoxide; EGFR-TKI, epidermal growth factor receptor tyrosine kinase inhibitor; EDTA·2Na, ethylenediaminetetraacetic acid disodium salt; FA, formic acid; HQC, high quality control; HS, human serum albumin; IS, inner standard; IPA, isopropanol; Cys, L-cysteine; LLE, liquid–liquid extraction; LLOQ, lower limit of quantitation; LQC, low quality control; MeOH, methanol; MQC, middle quality control; MWCO, molecular weight cut-off; NSCLCs, non-small-cell lung carcinomas; PFS, progression-free survival; RE, relative error; RSD, relative standard deviation; CMC·Na, sodium carboxymethyl cellulose; SD rats, Sprague–Dawley rats; UPLC-MS/MS, ultra-performance liquid chromatography–tandem mass spectrometer.
